# Effect of Atmospheric Corrosion on the Mechanical Properties of SAE 1020 Structural Steel

**DOI:** 10.3390/ma11040591

**Published:** 2018-04-11

**Authors:** Carola Martínez, Francisco Briones, María Villarroel, Rosa Vera

**Affiliations:** 1 Laboratorio de Corrosión, Instituto de Química, Pontificia Universidad Católica de Valparaíso, Av. Universidad 330, Valparaíso 3100000, Chile; maria.villarroel.m@pucv.cl (M.V.); rosa.vera@pucv.cl (R.V.); 2 Escuela de Ingeniería Mecánica, Pontificia Universidad Católica de Valparaíso, Los Carrera 01567, Quilpué 2430120, Chile; francisco.briones.cgl@gmail.com

**Keywords:** stress-strain test, structural steel, atmospheric corrosion, atmospheric parameters, XRD analysis

## Abstract

Resistance to atmospheric corrosion in different environments located in Chile and the corrosion’s effect on the mechanical properties of SAE 1020 steel were studied. Atmospheric corrosivity categories at each station under study were determined. These categories were C2, for Laja; C3 and C4, for the Arica and Antarctic stations, respectively; and the most aggressive, C5 and higher at Quintero. These specific environments significantly influenced the mechanical responses of steel exposed for 36 months. Rupture elongation, the modulus of toughness, ultimate tensile strength, and hardness of the material all decreased as a function of environmental atmospheric aggressiveness. Lowered ductility is the result of the increased corrosion rate due to the high deposition of chlorides. This is due to the morphology of material degradation, which consequently occurs as pores, microstrains, and other defects that promote early rupture of the steel.

## 1. Introduction

Atmospheric corrosion is a topic of worldwide concern, due to its influence on the shelf life of structural materials. High economic costs are associated with corrosion, due to the different approaches that each country must take to maintain materials and prevent corrosion [1]. This is most important in areas where the level of environmental aggressiveness is high. The conventional atmospheric parameters that may lead to metal corrosion are temperature, humidity, precipitation, solar radiation, wind speed and direction [2], and air contaminants, such as chlorides, sulfur dioxide, carbon dioxide, hydrogen sulfide, nitrogen oxides, etc. [3,4,5].

Carbon steel is widely used in engineering applications, due to its low cost, ease of manufacture, and properties that satisfy varying design requirements, particularly when environmental and strength conditions are not severe. When greater mechanical or corrosion resistance is needed, carbon steels are replaced by weathering or stainless steels [6,7,8], which incur additional costs. Atmospheric corrosion of steel can be divided into three stages: the wetting stage, the wet stage, and the drying out stage. Corrosion rate and modifications to the oxide layer are thus correlated with the number and frequency of the wet-dry cycles [9].

Atmospheric corrosion may lead to material structural decay, fatigue cracking, brittle fracture, and unstable failures [10,11], attributable to variations in surface roughness [12] resulting from heterogeneously distributed corrosion, diminished surface area, and the formation of pitting. In Chile, studies on atmospheric corrosion have been carried out, and have determined the influence of atmospheric parameters and pollutants [13,14,15,16,17,18,19]. In spite of the above, there still have not been many studies to determine just how drastic mechanical damage may be in the presence of different atmospheric conditions and exposure times for some zones in Chile [20].

The fundamental pillar for the design and selection of materials is the correct interpretation and application of mechanical properties, in order to facilitate economic maintenance decisions and to prolong structural lifetimes. One of the most relevant assays in adequate material selection is the stress-strain test. Therefore, this work presents a study of atmospheric corrosion’s influence on the mechanical properties of SAE 1020 steel, exposed to different climates in Chile for different exposure times.

## 2. Experiment

### 2.1. Sample Preparation 

SAE 1020 steel coupons (100 mm × 100 mm × 4 mm) were used as probes, placed at 45° angles and separated by plastic isolators. These were installed according to ISO standards 9223 to 9226 [21,22,23,24] at different locations in Chile, for 36 months, beginning in March 2010 [14,15]. The metals’ chemical compositions were analyzed by X-ray spectrometry, and are listed in Table 1.

Before performing the electrochemical and mechanical properties tests on the samples, corrosion products generated during exposure were removed from the coupons by submersion in an etching solution (HCl/(CH_2_)_6_N_4_) for 10 min, on each side of the coupons, at room temperature [25].

### 2.2. Environmental Conditions of Selected Locations

The locations of atmospheric stations were selected based on distinct atmospheric conditions: (i) Arica, close to the sea; (ii) Quintero, an industrial–marine zone; (iii) Laja, a rural zone; and (iv), Antarctic, close to the sea and low temperature. The deterioration from material corrosion was evaluated every 3 months, with corrosion loss measurements in triplicate (ASTM G50 [26]). Table 2 presents the geographical locations utilized during this study.

Devices were installed to take bimonthly measurements of chloride and sulfur dioxide content in the air. The wet candle method was used to measure atmospheric chlorides. This method consists of wrapping a strip of gauze around a glass tube, where the ends of the gauze are submerged in a solution of 10% glycerin in distilled water. The chlorides deposited from the air are measured via mercurimetry in the presence of diphenylcarbazone and bromophenol, and the results are expressed in mg Cl^−^ m^−2^ d^−1^ [23]. For measurements of SO_2_, the lead dioxide candle method was used: SO_2_ is deposited on gauze coated with PbO_2_, forming lead sulfate. This compound is solubilized in 5% Na_2_CO_3_, and sulfate ions are then measured gravimetrically by precipitation as BaSO_4_; the result is expressed in mg SO_2_ m^−2^ d^−1^ [23]. For those places that did not have the capability already, measurements of weather conditions—temperature, relative humidity, total rainfall, and wind speed (speed of the maximum burst occurring in a given day, and the mean value as the average considering every day)—were taken. The data on relative humidity and temperature were used to estimate time of wetness (TOW) [27].

Three environmental parameters were used to assess corrosivity categories: time of wetness (TOW), sulfur compounds based on sulfur dioxide (SO_2_), and airborne salinity contamination (Cl^−^). For these parameters, classification categories are defined as τ (TOW), P (SO_2_), and S (salinity (Cl^−^)).

### 2.3. Methods of Characterization of Corrosion Products Formed on the Surface of SAE 1020 Steel

The morphologies of the attacks on the metals were studied via optical microscopy (OM) with a Leica DM500 microscope, with and without polarized light, as well as via scanning electron microscopy (SEM) (Hitachi SU3500, Tokio, Japan). The phases formed on probe surfaces were determined via X-ray diffraction (X’PERT PRO, Cu-Kα radiation, 40 KV/40 mA graphite monochromator, 1° angle of incidence, nickel filter).

### 2.4. Methods Used for Determination of Electrochemical Behavior of SAE 1020 Steel 

Electrochemical measurements were made for SAE 1020 steel samples exposed for 36 months at the different stations, to determine the electrochemical response of the material 36 months post-exposure. For this reason, the corrosion products generated during the exposure time were removed from probes by submersion in an etching solution (HCl/(CH_2_)_6_N_4_) for 10 min on each side of the probe, at room temperature [25].

The electrochemical cell utilized a conventional arrangement of three electrodes, consisting of a working electrode, a saturated calomel reference electrode, and a Pt wire as a counter-electrode. The working electrode was prepared from an exposed steel sample mounted on epoxy resin, with 1.0 cm^2^ of total geometric area. The solution was prepared by dissolving analytic grade NaCl (Aldrich, Steinheim, Germany) in distilled water. The ion chloride (Cl^−^) concentration in the electrolyte was 0.1 M. The working electrode was immersed in the electrolyte, to stabilize the open circuit potential until the variation was less than 1 mV for 5 min before measurement. Potentiodynamic experiments started from the open circuit potential to anodic direction for 500 mV with a scanning rate 0.5 mV s^−1^.

### 2.5. Mechanical Testing of SAE 1020 Steel

**Figure 1:**
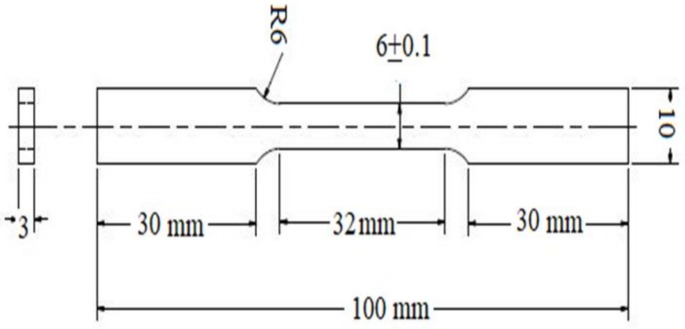
Schematic diagram of steel sample with dimensions.

From the exposed and cleaned surfaces of the steel coupons, samples were prepared for tension tests, according to ASTM E8 standards [28]. The dimensions of each probe are shown in Figure 1.

To evaluate mechanical properties, tensile tests were carried out on a Time Machine Inc. (Jinan, China) Universal Machine Model WDW-200E, with a capacity of 200 kN and digital data report, and a deformation speed on the order of 10^−3^ s^−1^. For each location and exposure time, probes were tested in triplicate. In addition, probes were tested for hardness in a Vicker microhardness tester, 300 g load, according to the ASTM E-92 standard [29]; each probe was tested 10 times. Specimen surfaces were roughened with 320–2500 grade SiC paper, and then polished with Al_2_O_3_ as an abrasive agent, with a particle size of 5 to 1 μm. This surface pretreatment was done for hardness determination.

## 3. Results and Discussion

### 3.1. Process of Atmospheric Corrosion on SAE 1020 Steel

**Figure 2:**
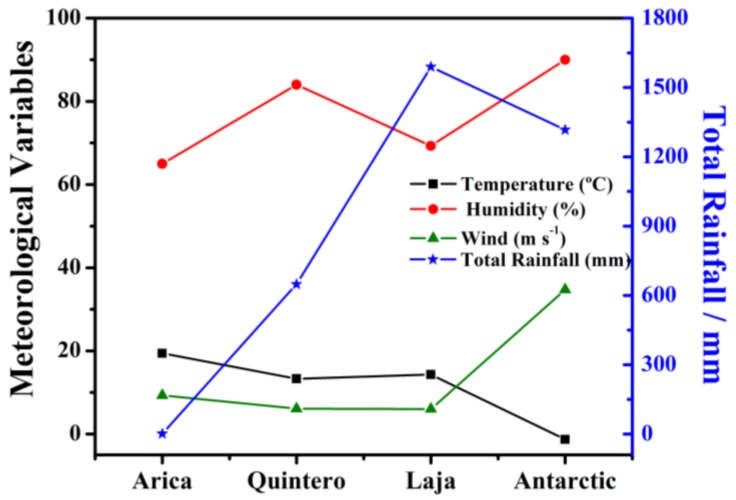
Weather variables measured at test stations.

Corrosion on SAE 1020 steel was determined from corrosion rate and corrosion loss during a 36-month exposure time. Electrochemical tests were performed on the exposed probes, to determine the variation of their electrochemical activity post-exposure. Figure 2 shows the mean annual values for each test station over the 36-month exposure time. The measured variables are temperature, relative humidity, total rainfall, and wind speed.

The temperature variation at different sites is due to latitude difference among the four study stations. Arica is in the north of Chile, a desert climate with abundant cloud cover, and has an average temperature of 19 °C. The temperature variation in Quintero is less, due to the moderating influence of the sea, hovering at around 13 °C; the relative humidity there, however, is higher for the same reason, at 85%, and it receives more abundant rainfall, at an average of 649 mm over the 36 month period. The Laja station is located in an Intermediate Depression zone with a predominately temperate climate, with an average temperature of 14 °C, a relative humidity of 69%, and precipitation of 1589 mm on average over 36 months. Finally, the Antarctic station presents glacial climates. This zone has average temperatures of −1.3 °C. Precipitation on the Antarctic Peninsula is difficult to determine, since (i) the majority of precipitation is in solid form, and when it does occur, strong winds make it difficult to take a representative measurement with traditional methods; and (ii), it is difficult to differentiate what is truly precipitation, or just snowdrift raised by the wind. The wind in the Antarctic reaches the greatest velocity, with respect to the other stations being studied, so precipitation measurements must be considered with these caveats. Precipitation measurements were at an average of 1317 mm over the period studied. It is important to note that an increase in temperature produces an increase in the kinetics of the electrochemical reactions; however, at the same time, it accelerates evaporation of electrolytes (necessary for electrochemical reactions to occur) away from metal surfaces [19].

Table 3 shows the atmospheric corrosivity categories for the 4 locations in this study, determined from meteorological conditions [21] discussed above. The zone with the highest category is the Quintero station (C5), while the lowest category is the rural Laja station (C2). The Arica and Antarctic stations, in spite of their large differences in latitude and climate, belong to category C3–C4.

**Figure 3:**
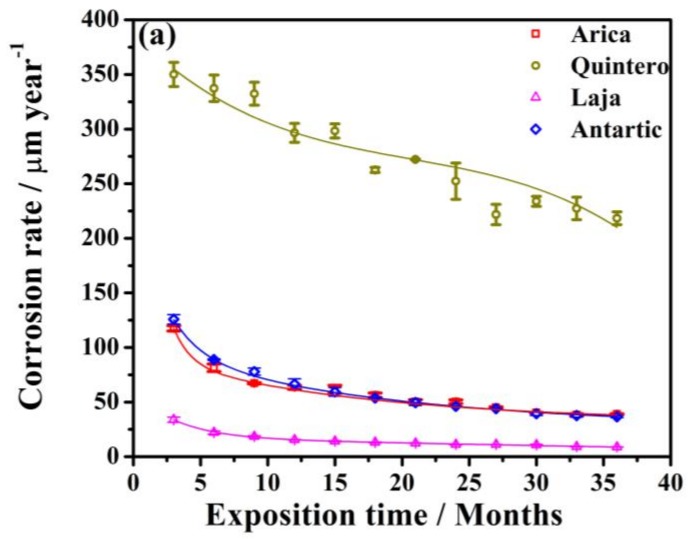

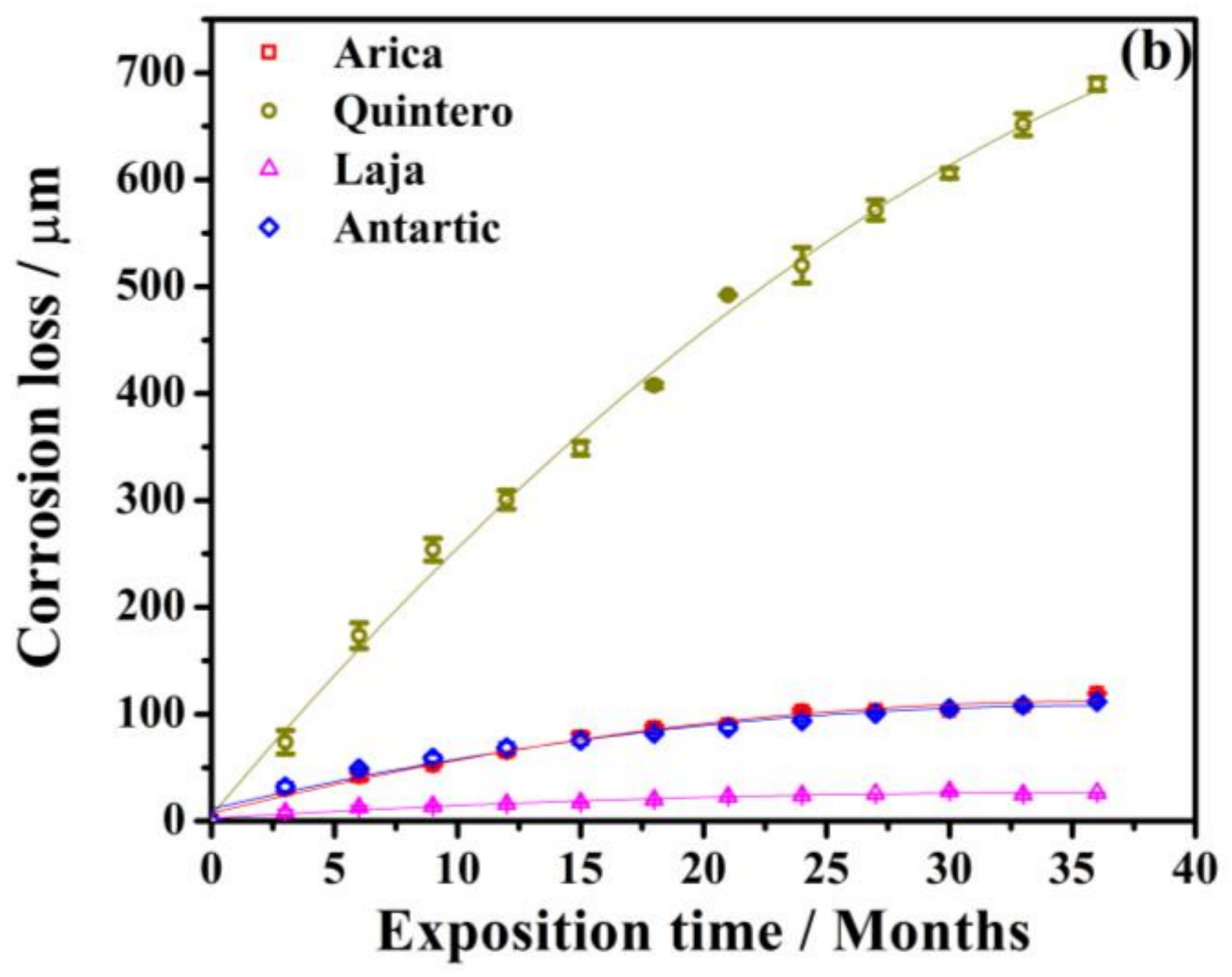
(**a**) Corrosion rate; (**b**) corrosion loss as a function of exposure time at different stations. The data were evaluated and averaged from triplicates.

To best establish steel behaviors in aggressive environments, Figure 3 presents variations in corrosion rate and corrosion loss, where steel corrosion rates from the four study stations are presented in Figure 3a. It can be observed that, as exposure time increases, the corrosion rate is reduced; this is attributed to the fact that as corrosion forms, it creates a protective layer [15]. This behavior follows an exponential curve, except at the Quintero station—this site had the greatest corrosion rate, and with the mix of industrial–marine environments, had an inconsistent behavior, based on the variations of these environmental conditions. This station again is found in the zone with the greatest amount of contamination, in terms of chloride ion and sulfur dioxide content (Table 3); this contamination probably has a synergistic effect on the corrosion response. Arica and Antarctic have lower corrosion rates compared to Quintero, although Arica has more chloride; however, it is the greater TOW at Quintero that is essential for the corrosion process to occur. If it were chloride content, Arica should have greater corrosion than the Antarctic. Here, the levels of corrosion are practically equal, since Antarctic has more TOW and SO_2_—this suggests that when the chloride ion is present, TOW is also needed, due to the hygroscopic nature of chloride and to thus maintain the activity. Furthermore, ice remaining on the steel surface maintains the metal–ice chloride ion interface. Indeed, analyzed ice samples contained about 60 mg Cl^−^ m^−2^ d^−1^. Ice also makes the product flat and smooth, which influences material deterioration. With respect to Laja, the lower rate of corrosion is due to lower chloride content.

Next, Figure 3b shows that the behavior at all stations follows a quadratic equation. The probe with the greatest corrosion loss was that at the Quintero station; the steel exposed in Laja, in a rural environment, had the least loss. Although the Laja rate could have been lower, the high precipitation there favors the corrosion process. Both the Arica and Antarctic stations had similar behavior, since both zones are close to the sea and are classified as marine, even though the ambient temperatures are quite distinct (Figure 2).

The categories, according to the aggressiveness levels [24,30] based on corrosion rate values, obtained after a year of exposure at each station, are as such: (i) Laja is classified as C2 (low aggressiveness), with rates of corrosion lower than 15.5 µm year^−1^; (ii) Arica and Antarctic are C4 (high), with values of 63.6 and 66.6 µm year^−1^, respectively; and (iii), the Quintero station, classified with very high aggressiveness, >C5, with a corrosion rate of 296.5 µm year^−1^. It is important to mention that, for most stations, the value obtained for the corrosion rate after only 3 months is greater than later months, given that the kinetics of the process is related to the formation of corrosion products, i.e., layers that slow down the corrosion rate. If the accumulated corrosion products are mostly insoluble, adherent, non-porous, and of a certain thickness, it is to be expected that the corrosion rate of the materials will diminish as a function of time [31,32]. This was the case in the majority of the stations.

Therefore, in decreasing order, the corrosion rates for SAE 1020 steel by location is:

Quintero > Antarctic = Arica > Laja.

### 3.2. Corrosion Products Formed on the Surface of SAE 1020 Steel

#### 3.2.1. Morphology of Corrosion Products

**Figure 4:**
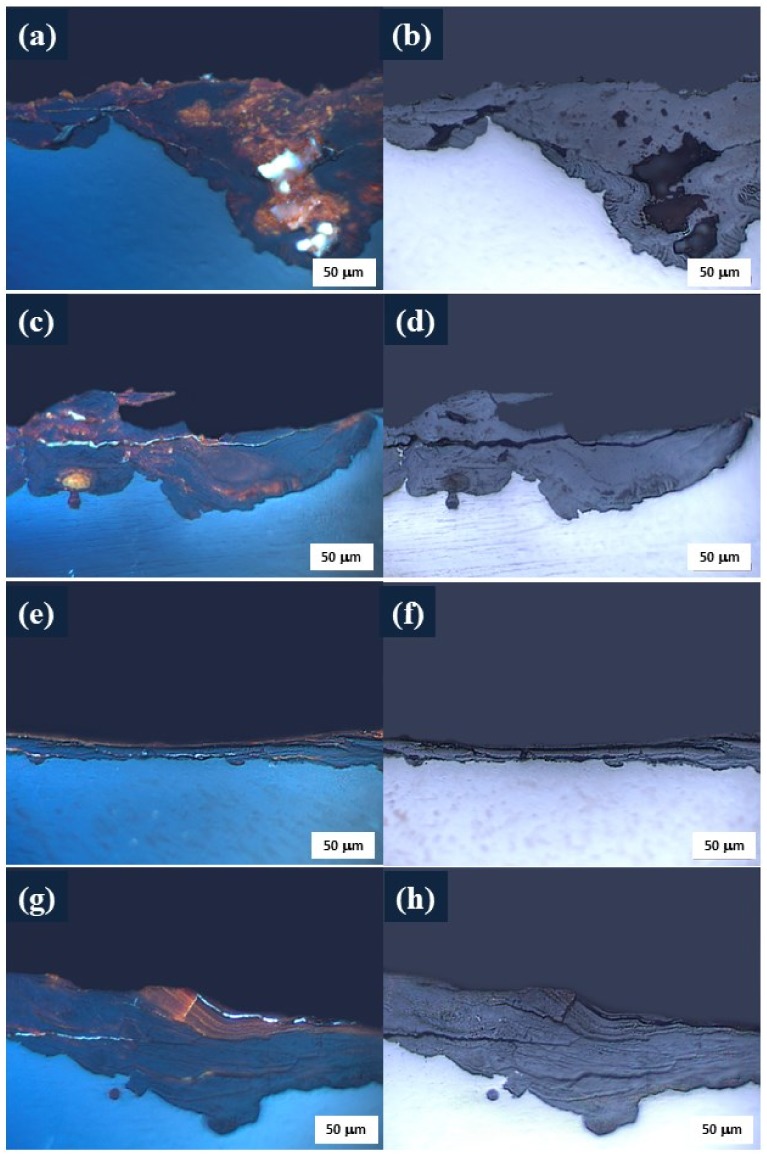
Optical micrograph of rust formed on the steels exposed for 36 months in different environments of Chile: (**a**,**b**), Arica; (**c**,**d**), Quintero; (**e**,**f**), Laja; and (**g**,**h**), Antarctic Stations. Magnification 200×.

Figure 4 shows optical micrographs, with and without polarized light, for rust formed on steel surfaces exposed at different locations. In the polarized light images (Figure 4a,c,e,g) two sub-layers can be seen in the oxide film: a colorless inner layer (dark gray) and an orange-brown outer layer. This phenomenon is also observed in weathering steels [33]. By observing films on the surface, with and without polarized light, both the formed layers and the depth of cracks and defects can be differentiated; for example, the brighter areas correspond to cracks or deeper pores in the formed film, while the more colored areas reveal whether the corrosion product is superficial or is more compact, an effect that cannot be detected in non-polarized light images (Figure 4b,d,f,h).

The corrosion rates between the Arica and Antarctic stations are practically the same, since both are classified as marine environments and as C3–C4 [21,24]. However, corrosion product films from the Arica station are more porous and heterogeneously formed; there, localized corrosion in certain spots is attributable to the inclusion of chloride ions. It is typical for atmospheres with higher chloride content to develop corrosion layers with many pores. In contrast, the film formed in Antarctic is apparently formed in layers (Figure 4g,h). The inner part of the film is more compact, and also has cracks parallel to the surface of the steel, and no observable corrosion product detachment. These cracks are attributable to the accumulation of soluble salts between sub-layers, which may change their state with temperature variations (corresponding to temperatures below 0 °C), which would cause the oxide layer to rupture [34].

In the case of the specimen located at the Laja station, which had the lowest corrosion rate, a thin film of corrosion product fluctuated between 14 and 29 μm thickness. This is attributed to more homogeneous corrosion (Figure 4e,f).

**Figure 5:**
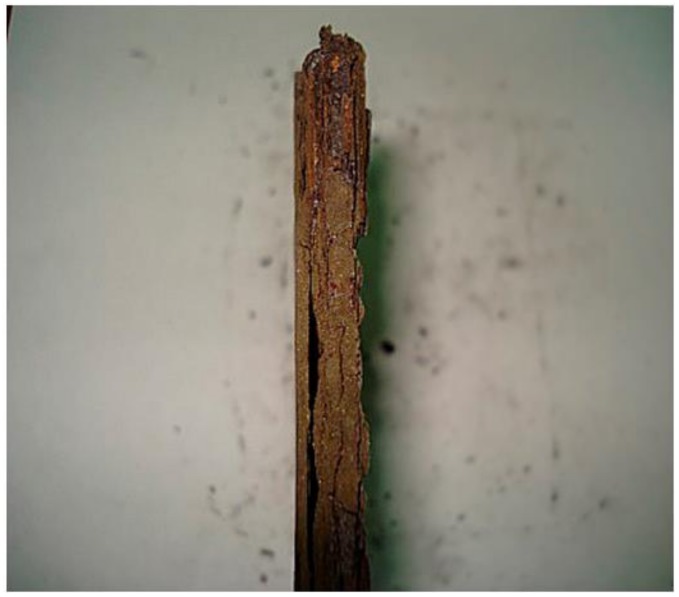
Optical macrograph of the exfoliation present in the exposed probe at the Quintero station after 36 months of exposure.

The film formed on the specimen located at Quintero (highest corrosion rate) did not differ greatly in thickness, as expected; rather, the formed corrosion products were continuously detached from the surface via exfoliation (Figure 5). This phenomenon is not seen in the exposed steel of Arica, Laja, or Antarctic. Corrosion exfoliation does not diminish over time, but rather begins on the surface, with corrosion products increasing in volume and producing ruptures in the film. One example of this cracking is clearly visible in Figure 4c,d. Although there is a cracked area in the Antarctic film, the crack is not deep, and does not cause corrosion products to peel off, as in the case of Quintero.

**Figure 6:**
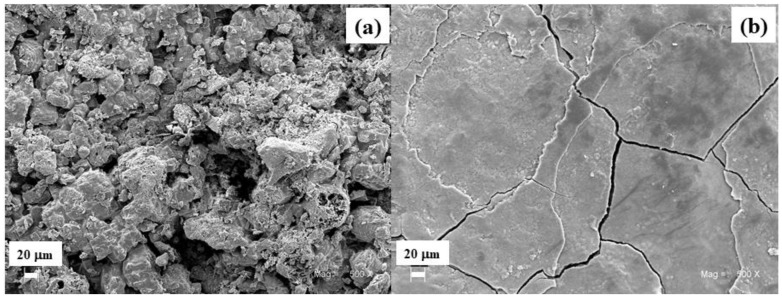
SEM images of corrosion products exposed for 36 months at the stations of (**a**) Arica and (**b**) Antarctic. Magnification 500×.

The different surface films formed on specimens in Arica and Antarctic were observed using SEM imaging (Figure 6). The corrosion products formed at the Arica station are irregular, less compact, and do not provide a layer to slow down corrosion rates, due to reduced penetration of aggressive corrosion stimulators to the steel surface. Additionally, at that site, there are pores acting as active sites for the chloride ion to enter the metal and produce localized corrosion. In Antarctic, on the other hand, the film is made up of flat and smooth layers and is much more compact, although cracked, as mentioned above. This type of film is produced due to ice sheets deposited on the specimens [13,34]. Differences in corrosion product porosity as a function of chloride ion concentration has been described in the literature before, e.g., Morcillo et al. [35]. That study determined that the structure of corrosion product layers formed on steel surfaces may result in two extreme situations, ranging from the formation of a “consolidated oxide layer” (at relatively low salinity) to the formation of highly imperfect, cracking, or exfoliated thick oxide layers, which are easily detached from the residual base steel (at very high salinity).

#### 3.2.2. X-ray Diffraction Analysis

**Figure 7:**
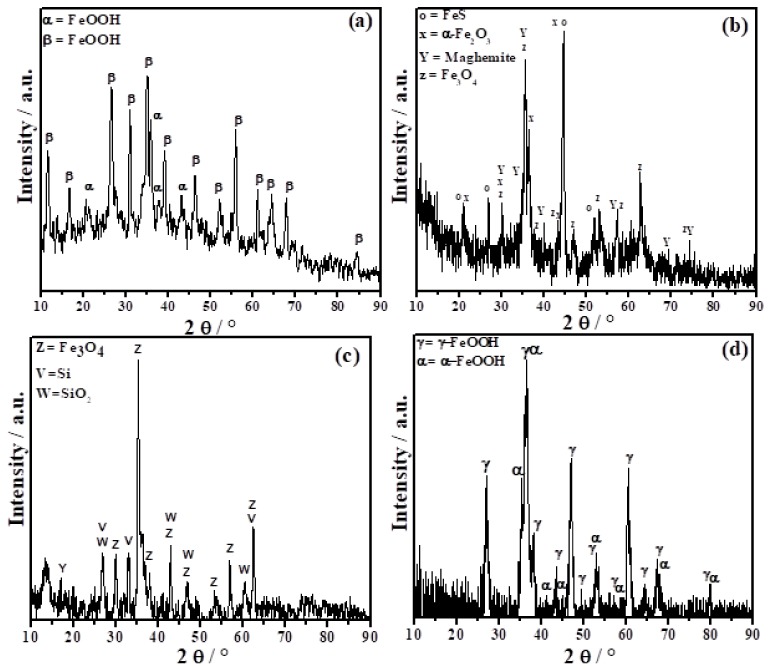
Diffraction patterns for corrosion products after 1 year of exposure. (**a**) Arica, (**b**) Quintero, (**c**) Laja, and (**d**) Antarctic stations.

The corrosion products most commonly found in atmospheric exposures of steel are lepidocrocite (gamma-FeOOH) and goethite (alpha–FeOOH), though in marine atmospheres, these are akaganeite (beta–FeOOH) and magnetite (Fe_3_O_4_). The formation mechanisms and the identification of these phases in corrosion layers have been, and continue to be, the cause of considerable controversy [36]. In this study, corrosion products formed after 1 year of exposure were determined by X-ray diffraction (Figure 7), and showed clear differences in the phases formed and deposited on the surface of the material depending on the exposure zone.

The corrosion sample phases at the Arica station, in a marine environment, were mainly a mixture of oxyhydroxides, including Akagaenite (beta-FeOOH, formed in environments with high concentrations of chloride ions (Cl^−^) [37]); and as a minority phase, goethite (alpha-FeOOH, a stable phase). At the Quintero station, a greater number of products formed due to the mixture of industrial–marine environments. There were three types of oxides and FeS, attributed to the high amount of sulfur dioxide (SO_2_) present in the environment (26.5 mg m^−2^ d^−1^ of SO_2_ observed). The phases formed in this case were: alpha-Fe_2_O_3_, 92%; iron sulfide (FeS), 3%; maghemite, 4%; and Fe_3_O_4_, 1%. The specimens located at Laja formed Fe_3_O_4_, with Si and SiO_2_ deposited on the surface (Si is present in the dirt). Finally, in the case of the Antarctic station, the corrosion products identified were a mixture of gamma-FeOOH and alpha-FeOOH. As can be seen in Figure 7, although the corrosion loss and corrosion rate of the stations located in Arica and Antarctic are practically the same, the phases formed are different; this can be attributed to the difference in the concentration of Cl^−^ (see Table 3), which is known to facilitate the formation of beta-FeOOH [38,39]. Additionally, since the oxyhydroxide akaganeite is non-protective [40], localized corrosion and porous film occurs on the steel surfaces at Arica. In Antarctic, on the other hand, gamma-FeOOH is the main component of the rust layer formed on steel, which is transformed into alpha-FeOOH [37,41]. Morcillo et al. [34] and J. F. Marco et al. [13] report the formation of gamma-FeOOH, alpha-FeOOH, and maghemite on the surface of exposed steel specimens in Antarctic, although in this case the transformation from alpha-FeOOH to maghemite is not observed in X-ray diffraction.

### 3.3. Electrochemical Behavior after 36 Months of Exposure

**Figure 8:**
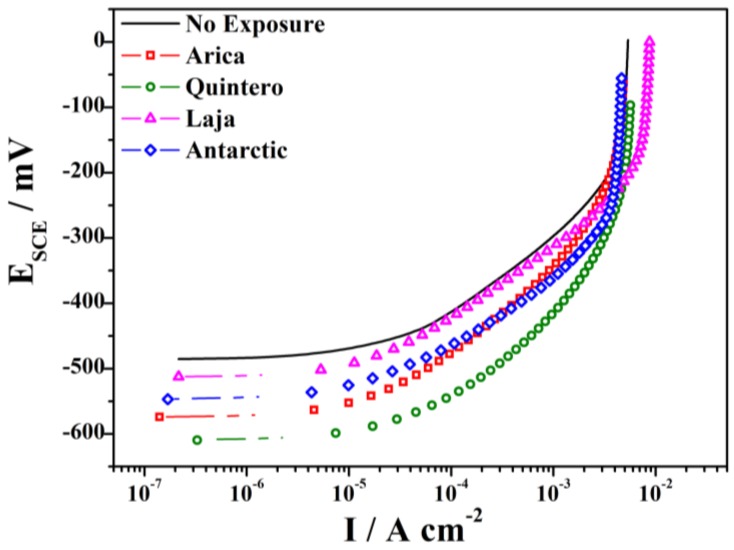
Anodic curves of SAE 1020 steel exposed for 36 months in different areas of Chile.

Figure 8 shows the electrochemical measurements of the steel exposed at different stations after 36 months. Corrosion products were removed from the specimens, and the open-circuit potential was stabilized for 5 min (i.e., the potential difference remained below 1 mV for 5 min) before the anodic curve of each sample was taken.

As can be seen in Figure 8, the shape of the anodic curves is similar; however, the starting open circuit potentials of the curves differ (Table 4), and some tend to be more negative than others, depending on the amount of atmospheric corrosion damage to the material. The increase of the anodic current is the result of increased real area of the sample, indicative of increased atmospheric corrosion rates creating rougher surfaces and bigger surface areas. Table 4 also shows the values of dissolution current densities at a constant potential of −400 mV. Results are compared with non-exposed steel (as a control), which corroborates lower damage to steel at the Laja station (1.6 × 10^−4^ A cm^−2^) and higher damage to material at the Quintero station (12 × 10^−4^ A cm^−2^). These data are consistent with those obtained from the corrosion rate.

### 3.4. Mechanical Properties of SAE 1020 Steel Post-Exposure 

**Figure 9:**
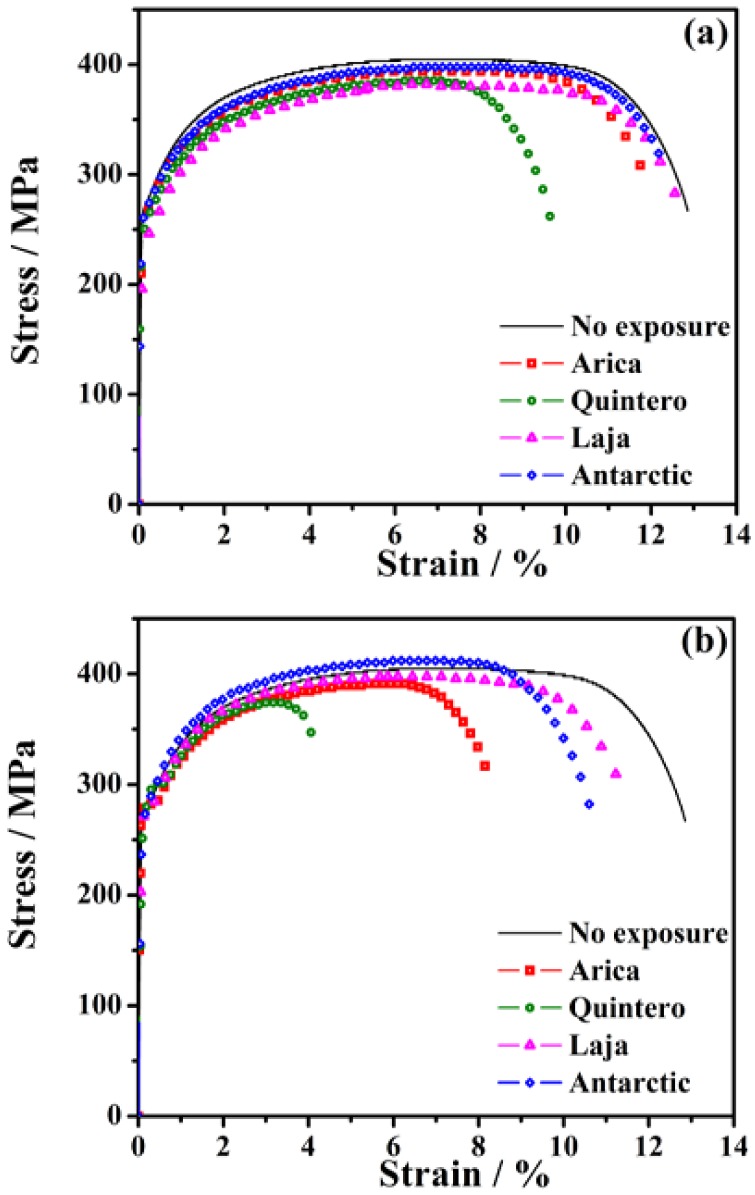
Stress-strain curves of the steel specimens exposed in different stations after (**a**) 6 months and (**b**) 36 months of exposure.

The mechanical characterization of SAE 1020 steel exposed at different locations in Chile was determined through the most commonly used method in the study of mechanical properties: the tensile test [9,12,42,43]. The traction curves of the different specimens exposed along Chile are shown in Figure 9.

At 6 months of exposure (Figure 9a), only the specimen exposed at Quintero presents a noticeable decrease in the mechanical properties of the material. For example, material from that location has the lowest percentage of deformation at rupture (ε_R_), a parameter associated with the ductility of the material, which is understood as the maximum amount of plastic deformation that a material is able to withstand before breaking. The fracture of the material may be a result of the pitting corrosion that developed very rapidly at the beginning, and the subsequent propagation of cracks. It is worth noting that this station has the highest corrosion rate, most likely due to exfoliation and thus loss of formed corrosion products. The removal of the corrosion products leaves the formation of pores, micro-cracks, abrasions, among others, on the surface of the steel, which inhibit elongation capacity and facilitate the early rupture of the material.

At 36 months (Figure 9b) there is a greater difference in the response of steel to uniaxial load application, depending on the station of exposure. Decreased ductility is the result of a decreased profile (cross-section, including pit depths), and thus the decreased area of tensile samples, due to corrosion losses. This is observed, for example, at the Laja station, where the formed corrosion product film is thinner and more homogeneous, and therefore not producing greater amounts of pitting on the surface of the material. It is known that increased pitting of the material causes greater concentrators of stress. The same phenomenon is observed from the Antarctic station, where there are no significant changes in ductility. In contrast, in Arica, which had the same corrosion rate as Antarctic but where the corrosion product film was not formed homogeneously, ductility decreases by almost 40%. M. Okayasu et al. [44], through simulations, found that areas near pitting corrosion have high levels of stress. This comparison reveals that as corrosion increases, so the corresponding elongation of the steel decreases before failure.

Another important parameter for steel assays is the ultimate tensile strength (UTS) of the material. This is the maximum possible tensile stress, and at this level of stress, the central section of the specimen begins to decrease in cross-sectional area. In the results obtained, there is little change in the overall ultimate strength of the alloy by location. That said, there are differences in UTS values, due to the heterogeneity inherent in surface properties and microstructure produced through atmospheric corrosion. The UTS in the Quintero probe decreased more compared to the other stations under study, and overall UTS values decreased, with the exception of the specimen located in Laja (rural environment); UTS there increased with respect to the unexposed specimen. This anomaly in the behavior of a specimen located at Laja had also been observed in previous studies on Charpy impact toughness measurements [20]. There, the increase in impact toughness was attributed to the existence of more homogeneous corrosion products, not localized corrosion. As mentioned above, mechanical properties directly depend on surface conditions (microgeometric and physicochemical). D. Novovic [45] reported that cracks caused by mechanical stresses, and their subsequent progression, generally begin in the most superficial layers of the material, since these layers experience the greatest loads.

Considering the main characteristic of this steel is its toughness, the area under the curve (Equation (1)) of the tensile strength test can be used to determine the modulus of toughness of the material at low strain velocity (Table 5).

(1) $$U_{t}\;=\;\int_{0}^{\varepsilon f}\sigma \;d\varepsilon$$

The term “modulus” is used because the units of deformation energy per unit volume area Nm m^−3^, are equivalent to N m^−2^ (MPa), the same unit as the stress or modulus of elasticity. The values are correlated with site and exposure time. In the most aggressive environments, toughness decreases considerably, up to 70% in the case of the material at the Quintero station.

Hardness values were obtained at exposure times of 6 and 36 months for different stations. The corrosion products were removed, and specimens were polished with Al_2_O_3_ with 1 μm grain size to obtain smooth surfaces free of porosity. Even considering this condition, on the surface of the material, variations in hardness measurements were observed. There is a decrease in resistance to steel indentation, in correlation with the aggressiveness of the environment in which specimens were exposed. Of note, the relationship between hardness and UTS is not as in many other materials [46]. Similar behavior was reported by Regab et al. [11], attributing this phenomenon of resistance to indentation decreasing, due to the formation of micro-pitting and micro-cracks, bulky corrosion products, and intergranular attacks. Therefore, surface defects, while affecting the hardness of steel, do not necessarily proportionally affect other properties based on the strength of the total material. As such, the familiar correlation between the UTS of steels and their hardness value may not be maintained.

**Figure 10:**
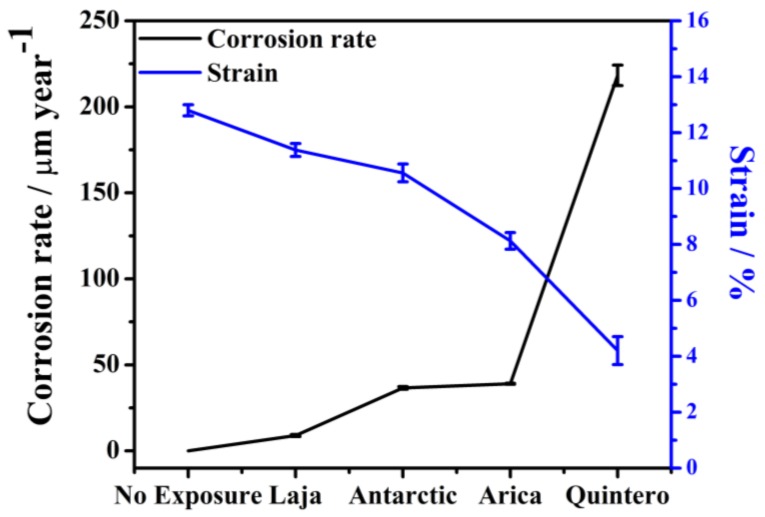
Relationship between corrosion rate and strain of exposed steel in different locations after 36 months of exposure.

The most evident effect is the decrease in steel ductility as a function of corrosion rate, as shown in Figure 10. In general, increased corrosion rate, environment aggressiveness, and material deterioration decreased ductility. Yuan et al. [47] reported that, as specimen thickness decreases in stress-strain tests, total elongation decreases.

Greater quantities of corrosion products affect the surface of the material and the loss of material; for example, in the specimen exposed at Quintero, corrosion caused exfoliation of the material, which directly affected the ductility of the material, due to thickness reduction. Moreover, in chloride attacks, the random formation of pits causes variation in steel mechanical properties, related not only to mass loss, but also to pit depth and extension. Although a similar decrease in corrosion loss was observed in the Antarctic and Arica stations, the mechanical properties of each were dissimilar after 36 months of exposure: smoother surfaces were obtained in Antarctic, which provided less probability of stress concentration; whereas rougher surfaces developed in Arica, with localized corrosion, such as pitting.

## 4. Conclusions

The classification of the corrosivity of the test stations based on environmental data coincides with the corrosion rates in all the stations under study. The decreasing order of corrosion rates for SAE 1020 steel is: Quintero > Antarctic = Arica > Laja.

This study has confirmed the synergy between sulfur dioxide (SO_2_) and chloride ion (Cl^−^) pollutants that originate various Fe oxides and generate greater deterioration in steel, i.e., at Quintero, with a C5 corrosivity category. For the Arica and Antarctic stations, although they were in same corrosivity category (C3–C4), the products formed on their surfaces are dissimilar, due to differences in Cl^−^ ion concentrations and temperature. The corrosion products formed at the Arica station are irregular and less compact, while those from the Antarctic were flat, smooth, and had much more compact layers—although, as mentioned above and shown in the SEM images, cracks were present. The most homogeneous film formed on any steel surface was in a rural environment, with corrosivity category C2 (Laja station).

In electrochemical assays, at higher corrosion rates, the starting potential is shifted towards more negative values, compared to non-exposed steel, as is the case with the Quintero station, which varies by −125 mV. In contrast, the sample with the slowest corrosion rate only varies in potential by −27 mV, at the Laja station.

The decrease of ductility is governed by a decrease in the sample cross-section, including pit depths, formed during exposure. The degree of degradation was lowest for the steel exposed at the Laja station, while the specimen exposed at Quintero (C5 category) showed the greatest loss in mechanical properties as a result of exfoliation, e.g., the ruptured deformation and toughness in that specimen decreased by 70%.

Chloride ions are most responsible for decrease in material ductility, as demonstrated by the mechanical responses of specimens exposed at the Arica and Antarctic stations. Although their aggressiveness classifications were the same, a greater presence of chloride ions caused greater material degradation at the Arica station.

## Figures and Tables

**Table 1:** Chemical composition of tested steels.

C	Mn	P	S	Si	Cr	Ni	Mo	Cu	V	Ti	W
0.098	0.28	0.012	0.015	0.15	0.03	0.04	0.07	0.03	<0.01	<0.01	<0.01

**Table 2:** Geographic locations of atmospheric stations.

Station	Latitude	Longitude	Sea Distance (m)	Sea Level (m a.s.l.)
Arica	18.47775 LS	70.320769 LO	579	20
Quintero	32.752625 LS	71.484508 LO	70	14
Laja	37.189947 LS	72.539644 LO	78000	118
Antarctic	63.320833 LS	57.899722 LO	100	10

**Table 3:** Atmospheric corrosivity categories (AC) of the distinct stations, based on pollution classification due to Time of Wetness (τ), airborne salinity contamination (Cl^−^) and sulfur compounds based on sulfur dioxide (SO_2_) concentration [14].

Station	TOW (%)	τ	mg m^−^^2^ d^−^^1^	Cl^−^	mg m^−^^2^ d^−^^1^	SO_2_	AC
Arica	6.3	τ3	125.6	S 2	5.0	P 0	3–4
Quintero	79.3	τ5	81.9	S 2	26.5	P 1	5
Laja	24.3	τ3	3.6	S 0	4.9	P 0	2
Antarctic	9.3	τ3	63.2	S 2	7.2	P 0	3–4

**Table 4:** Electrochemical parameters of the samples exposed for 36 months at different stations, obtained from the anodic polarization curve.

Sample	Potential (mV)	Dissolution Current Density at −400 mV (A cm^−2^)
No Exposure	−486.5	1.3 × 10^−4^
Arica	−574.7	4.0 × 10^−4^
Quintero	−611.1	12 × 10^−4^
Laja	−515.0	1.6 × 10^−4^
Antarctic	−548.7	4.6 × 10^−4^

**Table 5:** Specified mechanical properties of tensile and hardness testing of SAE 1020 steel samples exposed to different environments at selected exposure times.

Sample		ε Rupture	UTS	Module of Toughness	Hardness
Station	Time (Months)	(%)	(Mpa)	(Nm m^−3^)	(HV_0.3_)
No Exposure	0	12.80 (100%)	405	4846 (100%)	148.7 (100%)
Arica	6	11.71 (91%)	394	4337 (89%)	141.5 (95%)
	36	8.13 (64%)	391	2951 (61%)	132.4 (89%)
Quintero	6	9.62 (75%)	385	3398 (70%)	134.8 (91%)
	36	4.20 (33%)	374	1385 (29%)	124.0 (83%)
Laja	6	12.66 (99%)	382	4499 (93%)	132.6 (89%)
	36	11.38 (89%)	397	4239 (87%)	118.5 (80%)
Antarctic	6	12.31 (96%)	398	4594 (95%)	135.8 (91%)
	36	10.56 (82%)	412	4039 (83%)	135.2 (91%)
